# Immune Checkpoint-Mediated Interactions Between Cancer and Immune Cells in Prostate Adenocarcinoma and Melanoma

**DOI:** 10.3389/fimmu.2018.01786

**Published:** 2018-07-31

**Authors:** Angela Rita Elia, Sara Caputo, Matteo Bellone

**Affiliations:** Cellular Immunology Unit, Department of Immunology, Transplantation and Infectious Diseases, San Raffaele Scientific Institute, Milan, Italy

**Keywords:** prostate cancer, melanoma, immunity, immune checkpoint, immunotherapy, cytotoxic T lymphocytes

## Abstract

Prostate adenocarcinoma (PCa) and melanoma are paradigmatic examples of tumors that are either poorly or highly sensitive to therapies based on monoclonal antibodies directed against regulatory pathways in T lymphocytes [i.e., immune checkpoint blockade (ICB)]. Yet, approximately 40% of melanoma patients are resistant or acquire resistance to ICB. What characterize the microenvironment of PCa and ICB-resistant melanoma are a scanty cytotoxic T cell infiltrate and a strong immune suppression, respectively. Here, we compare the tumor microenvironment in these two subgroups of cancer patients, focusing on some among the most represented immune checkpoint molecules: cytotoxic T lymphocyte-associated antigen-4, programmed death-1, lymphocyte activation gene-3, and T cell immunoglobulin and mucin-domain containing-3. We also report on several examples of crosstalk between cancer and immune cells that are mediated by inhibitory immune checkpoints and identify promising strategies aimed at overcoming ICB resistance both in PCa and melanoma.

## Introduction

Activated T lymphocytes require mechanisms that timely and properly shut them down to prevent excessive damage at the inflammation site. Inhibitory immune checkpoint molecules, such as cytotoxic T lymphocyte-associated antigen-4 (CTLA-4), programmed death-1 (PD-1), lymphocyte activation gene-3 (LAG-3), and T cell immunoglobulin and mucin-domain containing-3 (TIM-3), are progressively upregulated on activated T cells, and, by interacting with their ligands, switch inhibitory pathways on in T cells (1). Interactions between immune checkpoint molecules on T cells and their ligands on target cells may also signal in the latters, thus generating a crosstalk between T lymphocytes and other cells (2–4). These mechanisms are crucial for self-tolerance, but also represent the Achilles’ heel of cancer immunity, as ligands for inhibitory immune checkpoint molecules are expressed on neoplastic and other cells within the tumor microenvironment. In addition, a growing tumor may condition secondary lymphoid organs, thus limiting expansion of tumor-specific T cells (5).

Building on these evidences, monoclonal antibodies (mAbs) directed against regulatory pathways in T lymphocytes [i.e., immune checkpoint blockade (ICB) (6)] have been developed. Phase III clinical trials with anti-PD-1/programmed death-ligand 1 (PD-L1) or anti-CTLA-4 mAbs documented excellent efficacy, and ICB has been approved for the treatment of various solid and hematological malignancies (7). Because several inhibitory checkpoints act simultaneously, the combination of two or more mAbs can improve ICB therapeutic outcomes (8).

Although melanomas are generally sensitive to ICB, also because of their heavy cytotoxic T lymphocyte (CTL) infiltrate, approximately 40% of melanoma patients are resistant to ICB even when two mAbs are combined (9). ICB resistance was recently reviewed [e.g., Ref. (10, 11)]. Other tumors like prostate adenocarcinoma (PCa) are intrinsically resistant to ICB (12), and either anti-PD-1/PD-L1 or anti-CTLA-4 monotherapy did not impact PCa patients’ overall survival (13, 14). ICB resistance in PCa is attributed to tumor cell intrinsic mechanisms and a scanty immune infiltrate (15) dominated by macrophages. In addition, soon after ICB, immune cells upregulate other inhibitory molecules such as V-domain Ig suppressor of T cell activation [VISTA; (16)], a phenomenon not limited to PCa (17). Interestingly, orally available small molecules targeting both PD-L1 and VISTA are investigated in patients affected by advanced tumors (ClinicalTrials.gov Identifier: NCT02812875).

Therefore, melanoma and PCa epitomize two classes of ICB-resistant tumors, in which tumor cell-intrinsic mechanisms of ICB resistance associate with heavy but immunosuppressed or modest immune infiltrates, respectively. Thus, while in the former the combination of two or more ICB mAbs should succeed, in the latter strategies to improve tumor infiltration by CTLs will be needed to improve ICB sensitivity. We will analyze differences and similarities in ICB-resistant melanoma and PCa, focusing on immune checkpoint-mediated interactions between tumor and immune cells. We will also highlight strategies that might improve sensitivity to ICB.

## T Cell Exhaustion

Prolonged antigen exposure progressively impairs T cell proliferation and effector functions (18) through epigenetic mechanisms (19). In the early dysfunctional state, which is plastic and reprogrammable, CD8^+^ T cells express PD-1 and LAG-3 and low TIM-3 levels. Later on, T cells enter fixed exhaustion characterized by TIM-3 upregulation, and the additional expression of high CD38 and CD101 and low CD5 levels. The latter cells are no longer reprogrammable by ICB (20). Partially exhausted CD8^+^ T cells, expressing high levels of PD-1 and CTLA-4 predicted response to anti-PD-1 in metastatic melanoma patients (21). Because also activated T cells express PD-1, this molecule cannot be used as marker of exhaustion, which should instead be functionally defined (22). Additional exhaustion markers (e.g., 2B4 and TIGIT) cannot be discussed here because of space constraint.

Also CD4^+^ T cells undergo exhaustion (23), loosing helper function and releasing IL-10 (24). CTLA-4 on CD4^+^ Tregs is an additional mechanism of immune suppression in cancer (25).

## Cytotoxic T Lymphocyte-Associated Antigen-4

Cytotoxic T lymphocyte-associated antigen-4 has been the first immune checkpoint investigated in clinic. Because of higher affinity for CD80 and CD86 than CD28, CTLA-4 impairs T cell co-stimulation (26). Whereas CTLA-4 is expressed on activated effector T cells (27), it is constitutively expressed on Tregs and contributes to their immunosuppressive activity. Thus, anti-CTLA-4 mAbs mainly act in secondary lymphoid organs, also causing Treg depletion through antibody-dependent cellular toxicity (28).

IFN-γ signaling activates expression of CTLA-4 in melanoma cells, and after ipilimumab (anti-CTLA-4) treatment, human melanomas upregulated IFN-γ responsive genes, including CTLA-4, which associated with durable response (29). Thus, anti-CTLA-4 mAbs can directly affect melanoma cells (30). CTLA-4 on tumor cells might also act as local mechanism of immune escape. Of relevance, mutations in the IFN responsive genes associate with resistance to ICB (31). Restifo and collaborators found that also mutations in genes indirectly correlated to the IFN response (e.g., *APLNR*), profoundly affected *in vivo* sensitivity to both adoptive T cell therapy (ACT) and anti-CTLA-4 blockade (32). It is anticipated that investigations on such comprehensive lists of genes will identify new drugs overcoming ICB resistance.

An alternative strategy to increase sensitivity to ICB is to combine them with other therapeutic strategies, such as chemotherapy, hormonal therapy, vaccines, etc. (Tables 1 and 2). As an example, both in mice and humans, the combination of local chemotherapy and systemic ICB increased tumor infiltration by effector T cells, and clinical response rates (NCT01323517) (33). Others have shown that targeting myeloid-derived suppressor cells (MDSCs), which are relevant immunosuppressive populations in PCa (34, 35), with tyrosine kinase inhibitors increased sensitivity to ICB in castration-resistant PCa (36). Both in orthotopic melanoma and autochthonous PCa, even the combination of anti-CTLA-4 and anti-PD-1 exerted modest antitumor effects (37), and required the addition of fresh T cells (i.e., ACT) and minute amounts of TNF-α targeted to tumor-associated vessels to favor endothelial cell activation, tumor infiltration by fully effector T cells, and tumor debulking (38, 39). Interestingly, only this triple-combined treatment guaranteed a prolonged overall survival of the mice affected by autochthonous PCa, thus suggesting the treatment generated a potent tumor-specific memory response (37). Additional strategies can be implemented to favor access of both T cells and mAbs to the tumor (40).

**Table 1:** Clinical trials of immune checkpoint blockade (ICB) combined with other strategies in prostate adenocarcinoma (PCa).

Target	ICB drug	Partner drug	NCT number	Status
CTLA-4	Ipilimumab	Evofosfamide	NCT03098160	Recruiting
CTLA-4	Ipilimumab	Sipuleucel-T	NCT01804465	Recruiting
CTLA-4	Ipilimumab	Cryoimmunotherapy ciclophosphamide	NCT02423928	Recruiting
CTLA-4	Ipilimumab	PROSTVAC V/F	NCT02506114	Recruiting
PD-1	PDR001	NIS793^a^	NCT02947165	Recruiting
PD-1	M7824^b^	ALT-803^c^; NCB024360^d^	NCT03493945	Recruiting
PD-1	Nivolumab	PROSTVAC V/F	NCT02933255	Recruiting
PD-L1	MDI4736	Olaparib cedinarib	NCT02484404	Recruiting
CTLA-4 and PD-L1	Tremelimulab and durvalumab		NCT02788773	Recruiting
CTLA-4 and PD-L1	Tremelimulab and durvalumab	polyICLC^e^	NCT02643303	Recruiting
CTLA-4 and PD-1	Ipilimumab and nivolumab		NCT03061539	Recruiting
CTLA-4 and PD-1	Ipilimumab and REGN2810	Stereotactic body radiation	NCT03477864	Not yet recruiting
LAG-3 and PD-1	LAG525 and PDR001		NCT03365791	Recruiting

*Selected clinical trials combining ICB and/or other therapies in PCa*.

*^a^Anti-TGF-β monoclonal antibody*.

*^b^Bifunctional fusion protein consisting of an anti-programmed death-ligand 1 (PD-L1) antibody and the extracellular domain of TGF-β receptor type 2*.

*^c^IL-15/IL-15Rα superagonist complex*.

*^d^IDO1 inhibitor*.

*^e^Toll-like receptor agonist*.

**Table 2:** Clinical trials of immune checkpoint blockade (ICB) combined with other strategies in melanoma.

Target	ICB drug	Partner drug	NCT number	Status
CTLA-4	Ipilimumab	Dabrafenib	NCT01940809	Recruiting
CTLA-4	Ipilimumab	6MHP^a^ peptide vaccine	NCT02385669	Recruiting
PD-1		INT230-6^b^	NCT03058289	Recruiting
PD-1	Pembrolizumab	iMIQUIMOD^c^	NCT03276832	Recruiting
PD-1	Pembrolizumab	Dabrafenib; trametinib	NCT02130466	Recruiting
PD-1	Pembrolizumab	Navarixin^d^	NCT03473925	Recruiting
PD-1	Nivolumab	PD-L1/IDO peptide vaccine	NCT03047928	Recruiting
PD-1	Pembrolizumab	IMP321^e^	NCT02676869	Recruiting
PD-L1	Atzolizumab	RO719857^f^	NCT03289962	Recruiting
CTLA-4 and PD-1	Ipilimumab and nivolumab		NCT03354962	Not yet recruiting
CTLA-4 and PD-1	Ipilimumab and pembrolizumab	Aspirin^®^	NCT03396952	Recruiting
PD-1 and TIM-3	PDR001 and MBG453		NCT02608268	Recruiting
CTLA-4 and PDL-1	Durvalumab and tremelimumab	IMCgp100^g^	NCT02535078	Recruiting

*Selected clinical trials combining ICB and/or other therapies in melanoma*.

*^a^High-dose IFN-α2b*.

*^b^Supermolecular complex of cisplatin, vinblastine, and an amphiphilic penetration enhancer*.

*^c^Synthetic agent with immune response modifying activity*.

*^d^CXCR2 antagonist*.

*^e^LAG-3Ig fusion protein*.

*^f^Messenger RNA based individually personalized cancer vaccine*.

*^g^Soluble gp100-specific T cell receptor with anti-CD3 single chain antibody fragment*.

Overall, these data support the concept that several therapeutic strategies need to be combined to overcome ICB resistance.

## PD-1/PD-L1

Programmed death-1 is upregulated on T cells upon antigen recognition, and by interacting with either PD-L1 expressed on tumor, stromal and immune cells or PD-L2 expressed on myeloid cells, impairs T cell activation (41). An exhaustion-specific enhancer regulates PD-1 expression in T cells (42), and editing exhaustion-specific enhancers might improve the therapeutic efficacy of ACT. Similarly, blocking *de novo* DNA methylation in chronically stimulated CD8^+^ T cells allowed retention of their effector functions (43).

Programmed death-1 blockade with nivolumab, lambrolizumab, or pembrolizumab has led to relevant clinical benefits in cancer patients, mainly by rejuvenating cytotoxicity and cytokine secretion capability of T cells (44). However, as mentioned above, T cells undergoing fixed exhaustion are no longer reprogrammable by ICB. An interesting study compared the epigenetic regulation of tumor- or virus-specific T cells in melanoma-bearing mice. Only melanoma-infiltrating, tumor-specific T lymphocytes (TILs) upregulated PD-1, LAG-3, and TIM-3 and showed reduced TNF-α, IFN-γ, and IL-2 secretion ability when compared with virus-specific cells. Exhausted T cells displayed more accessible chromatin in proximity to PD-1 and LAG-3 gene promoters. Treatment with anti-PD-1 mAbs had a positive impact on effector functions of exhausted T cells and on tumor growth, but induced only limited changes in gene expression and chromatin accessibility (45). Similar findings have been reported in a transplantable model of PCa, in which tumor-specific CD8^+^ T cells showed *de novo* methylation in *Tcf7, Ccr7, Myc*, and *IFN-*γ genes, and impaired proliferation and effector functions that could not be restored by ICB. Only combination of decitabine, inhibiting the DNA methyltransferase DNMT3A, and anti-PD-1 mAbs re-established proliferation capability of exhausted T cells, thus resulting in delayed tumor growth (43).

Clinical trials evaluating the efficacy of pembrolizumab in combination with epigenetic drugs are ongoing in advanced melanoma patients (NCT03278665, NCT02816021, and NCT02437136). Also in PCa, PD-1 blockade is clinically investigated in combination with ipilimumab (NCT02601014), anti-PD-L1 (NCT03170960, NCT03061539), and other therapies including hormone, vaccine, and cryosurgery (NCT02787005, NCT02499835, and NCT02489357).

Programmed death-1 can be found expressed also on tumor cells, and PD-1 triggering on melanoma cells increases three-dimensional growth capability with concomitant activation of the mTOR pathway (3). Interestingly, treatment with BRAF and MEK inhibitors associated with increased frequency of PD-1^+^ tumor cells in melanoma patients, and PD-1 expression sensitized melanoma to PD-1 blockade in immunodeficient mice (46). The same authors also noticed a correlative expression of PD-1 and the stem cell marker Oct-4, thus linking PD-1 to cancer stem cells (46).

Also anti-PD-L1 mAbs may directly affect tumor cells by impacting tumor metabolism, reducing extracellular acidification, phosphorylation of mTOR, and glycolysis (4). *mTORC1* expression has been associated with PD-L1 expression in melanoma cells, and PD-L1^low^ cells showed decreased levels of *mTORC1*, and an altered autophagy pathway. Furthermore, treatment of immunodeficient mice with anti-PD-L1 mAbs delayed melanoma growth, reduced metastases, and prolonged animal survival (2). PD-L1 has also been found overexpressed in melanoma tumor-initiating cells, and the lack of PD-L1 significantly reduced the frequency of these cells in melanoma-bearing mice (47). Thus, interfering with the PD-1/PD-L1 axis may impact both tumor and immune cells.

## Lymphocyte Activation Gene-3

Lymphocyte activation gene-3 is closely related to CD4, is expressed on dysfunctional T cells (48), and TILs in melanoma patients express LAG-3 (49). Because LAG-3 binding to MHC class II molecules activates myeloid cells (50), and MHC class II can be expressed by melanoma cells (51), engagement of LAG-3 with MHC class II might provide a survival signal to tumor cells. LAG-3 also binds LSECtin and Galectin-3 (Gal-3) (49, 52) and associates with the CD3/TCR complex, thus impairs TCR signaling (18, 52). Conversely, LAG-3 binding on Tregs increases their immunosuppressive activity (53).

Lymphocyte activation gene-3 may synergize with other immune checkpoints, and the combination of anti-LAG-3 and anti-PD-1 resulted in more potent inhibition of murine tumor growth than single treatments (54). Anti-LAG-3 mAbs or LAG-3 fusion proteins are being tested in melanoma patients resistant to anti-PD-1/PD-L1 ICB as single agent (NCT01968109), or in combination to anti-PD-1 (NCT02676869).

Drake and collaborators originally reported that in PCa, tumor-specific CD4^+^ and CD8^+^ T cells rapidly upregulate LAG-3 upon *in vivo* antigen encounter. Treatment with anti-LAG-3 mAbs enhanced the number and effector function of tumor-specific CD8^+^ T cells in TRAMP mice, and delayed tumor growth (55). Also Tregs in human PCa lesions upregulate both CTLA-4 and LAG-3 (56). The latter finding has been challenged by recent data showing low expression of LAG-3 in Tregs infiltrating PCa lesions (57). Further investigation is needed to better define the role of LAG-3 in T cell exhaustion and/or Treg function in PCa. One clinical trial is ongoing that investigates efficacy of anti-LAG-3 mAbs in combination with anti-PD-1 in castration-resistant PCa (NCT03365791).

## T Cell Immunoglobulin and Mucin-Domain Containing-3

Programmed death-1 expression in TILs is often associated with TIM-3, and its transient or persistent expression relates to short or chronic antigen stimulations, respectively (58). Indeed, PD-1^+^TIM-3^+^ T cells are functionally more exhausted than PD-1^+^TIM-3^−/low^ T cells (59), and TIM-3 can be considered a marker of terminally differentiated T cells.

T cell immunoglobulin and mucin-domain containing-3 is expressed on dysfunctional, tumor-specific CD8^+^ T cells in melanoma (60) and PCa patients (61), and in ipilimumab-treated melanoma patients, increased expression and frequency of TIM-3 and PD-1 on both peripheral NK and T cells associated with poor prognosis (62). Correlative data on TIM-3 in PCa patients are conflicting. Whereas one report showed that high TIM-3 expression on PCa cells predicted short recurrence-free and progression-free survival in chemotherapy and radiotherapy naïve PCa patients (63), others found that negative TIM-3 expression was an independent prognostic factor of poor prognosis in advanced metastatic PCa (64). Outcome differences might be brought back to the different subpopulations of PCa patients analyzed in the two studies. The latter also showed that silencing TIM-3 in PCa cell lines reduced tumor cell proliferation and invasion *in vitro* (63), thus, suggesting that TIM-3 has a functional role in PCa cells. Interestingly, the combined targeting of TIM-3 and PD-1 pathways is more effective in controlling tumor growth than targeting either pathway alone (59).

Mechanistically, the interaction between TIM-3 on T cells and one of its ligands [i.e., Galectin-9 (Gal-9)] triggers cell death in effector T cells (65). Ceacam-1, an additional TIM-3 ligand, is co-expressed on exhausted T cells, can bind TIM-3 both in *cis* and *trans*, and both interactions drive the inhibitory function of TIM-3 (66). TIM-3 also enhances FoxP3^+^ Tregs inhibitory functions (59), and is expressed and upregulated upon activation on human NK cells. In contrast to effector T cells, Gal-9-mediated TIM-3 triggering in NK cells induces IFN-γ production (67). Interestingly, it has been shown that MHC class I downregulation or deficiency in mouse tumors induces upregulation of PD-1 and TIM-3 on NK cells and their exhaustion. PD-1^+^TIM-3^+^ NK cells were also found in human melanoma samples, and correlated with low HLA expression (68). Because *in vitro*, TIM-3 blockade reversed NK cell exhaustion (69), it will be interesting to investigate the *in vivo* effects of mAbs against both PD-1 and TIM-3 on NK cells.

While TIM-3 is higher and more precociously upregulated on tumor-associated dendritic cells (DCs) than on CD8^+^ T cells, its role in innate immunity is controversial (70). By interacting with phosphatidylserine, TIM-3 favors DC uptake of apoptotic cells and cross-presentation (71). Conversely, interaction of the alarmin high mobility group protein B1 with Tim-3 on DCs limits their release of pro-inflammatory cytokines, thus blunting type-1 immunity (72). TIM-3 is also expressed on tumor-associated macrophages (72), and TIM-3 negatively modulates the production of pro-inflammatory cytokines in human CD14^+^ monocytes (73). Finally, TIM-3 can suppress the antitumor immunity by promoting induction of MDSCs (74).

Clinical trials are investigating safety and tolerability of anti-TIM-3 mAbs given either alone (NCT03489343) or in combination with anti-PD-1 (NCT02817633 and NCT02608268) or anti-PD-L1 (NCT03099109) in cancer patients.

## Galectins

Apart from being ligands for LAG-3 and TIM-3, galectins also exert relevant pro-tumor functions (75). Increased expression of Gal-3 in melanoma lesions correlates with tumor progression (76), and Gal-3 activates NFAT1 (77), which also regulates IL-8 and MMP3 expression in melanoma cells, thus promoting a malignant phenotype (78). Gal-3 released by melanoma cells can also capture IFN-γ, thus reducing its antitumor activity (79). At odds, others reported that tumor cell expression of Gal-3 or myeloid cell expression of Gal-9 in melanoma lesions associated with a longer survival (80). The latter findings are counterintuitive and deserve further investigation.

Inhibiting Gal-3 together with anticancer vaccination restores the effector function of melanoma TILs (81). Therefore, Gal-3 not only contributes to melanoma tumor growth and metastasis but also dampens the antitumor immune response. Gal-3 inhibition is currently investigated in combination with ICB and vaccine in melanoma (NCT02575404, NCT02117362, and NCT01723813).

Galectin-3 is also expressed in PCa lesions, exerts direct pro-tumor and pro-metastatic functions, and correlates with biochemical recurrence (82). Indeed, administration of a Gal-3 inhibitor suppressed PCa lung metastasis (83).

Galectin-3 is a marker of cancer stem cells (84) and maintains stemness of carcinoma progenitor cells (85). In the TRAMP model, we found that PCa stem-like cells endowed with immunosuppressive activities express Gal-3 (86). We have also evidence that Gal-3 favors growth and metastasis of tumors generated by PCa stem-like cells (Caputo et al., manuscript in preparation). It will be interesting to investigate if Gal-3 also contributes to their immunosuppressive activity.

## Concluding Remarks

Inhibitory immune checkpoint triggering in TILs cripples cancer immune surveillance. As consequence of local inflammation, immune checkpoints are also upregulated on cancer cells, supporting tumor growth and aggressiveness. Thus, the effect of ICB goes beyond rescuing of exhausted/dysfunctional TILs and may directly impact tumor cells.

To overcome TIL exhaustion, several promising combined approaches are envisioned among many others: coupling two or more mAbs against immune checkpoints; increase tumor immunogenicity by exploiting conventional chemotherapy and targeted anticancer agents (87); transiently modify the tumor vasculature to favor T cell infiltration (88–90); combine additional immunotherapeutic approaches such as vaccines and ACT (37); abolish additional mechanisms of local immune suppression (91). Several high throughput analyses (e.g., methylomics and metabolomics) and microbiota sequencing will likely define novel areas of therapeutic intervention in the field of ICB. Finally, it will be essential to focus on adverse events that increase along with therapeutic efficacy (92).

## Author Contributions

AE, SC, and MB wrote and reviewed the manuscript.

## Conflict of Interest Statement

The authors declare that the research was conducted in the absence of any commercial or financial relationships that could be construed as a potential conflict of interest.
